# Dual Stream Long Short-Term Memory Feature Fusion Classifier for Surface Electromyography Gesture Recognition

**DOI:** 10.3390/s24113631

**Published:** 2024-06-04

**Authors:** Kexin Zhang, Francisco J. Badesa, Yinlong Liu, Manuel Ferre Pérez

**Affiliations:** 1 Centre for Automation and Robotics (CAR) UPM-CSIC, Universidad Politécnica de Madrid (UPM), 28006 Madrid, Spain; kexin.zhang@alumnos.upm.es (K.Z.); javier.badesa@upm.es (F.J.B.); 2 State Key Laboratory of Internet of Things for Smart City, University of Macao, Macao; yinlongliu@um.edu.mo

**Keywords:** deep learning, gesture recognition, dual stream LSTM, feature fusion

## Abstract

Gesture recognition using electromyography (EMG) signals has prevailed recently in the field of human–computer interactions for controlling intelligent prosthetics. Currently, machine learning and deep learning are the two most commonly employed methods for classifying hand gestures. Despite traditional machine learning methods already achieving impressive performance, it is still a huge amount of work to carry out feature extraction manually. The existing deep learning methods utilize complex neural network architectures to achieve higher accuracy, which will suffer from overfitting, insufficient adaptability, and low recognition accuracy. To improve the existing phenomenon, a novel lightweight model named dual stream LSTM feature fusion classifier is proposed based on the concatenation of five time-domain features of EMG signals and raw data, which are both processed with one-dimensional convolutional neural networks and LSTM layers to carry out the classification. The proposed method can effectively capture global features of EMG signals using a simple architecture, which means less computational cost. An experiment is conducted on a public DB1 dataset with 52 gestures, and each of the 27 subjects repeats every gesture 10 times. The accuracy rate achieved by the model is 89.66%, which is comparable to that achieved by more complex deep learning neural networks, and the inference time for each gesture is 87.6 ms, which can also be implied in a real-time control system. The proposed model is validated using a subject-wise experiment on 10 out of the 40 subjects in the DB2 dataset, achieving a mean accuracy of 91.74%. This is illustrated by its ability to fuse time-domain features and raw data to extract more effective information from the sEMG signal and select an appropriate, efficient, lightweight network to enhance the recognition results.

## 1. Introduction

Electromyography (EMG) is a biomedical signal that is capable of reflecting muscle activation and detecting human movement intention. EMG offers distinct advantages in robot control and gesture recognition over traditional motion capture or kinematic and kinetic variables. EMG can detect muscle activation before actual muscle tension is produced, allowing for real-time robotic systems control, reducing computational lag, and facilitating synchronized motion and feedback. For instance, Pratt et al. [1] demonstrated how EMG’s advanced signal detection technology can enhance the operation of a robotic exoskeleton to achieve near real-time driving of the device. Additionally, EMG can detect certain muscle activations that do not necessarily cause movement but can indicate joint stiffness or other changes in the internal state. Borzelli et al. [2] explored how these signals can be used to modulate joint impedance without producing actual movement, which is movement intention information that cannot be obtained through kinetic or kinematic data. Thus, EMG not only enhances the responsiveness of robotic systems but also deepens our understanding of user intentions. These capabilities make EMG an invaluable tool for advancing the field of robot control and improving gesture recognition technologies. In reality, the EMG signal represents the electrical activity of a muscle’s motor units, which consists of two types: surface EMG (sEMG) [3] and intramuscular EMG (iEMG). Recently, the utilization of biological signals that contain a significant amount of human motion intention information has emerged as a promising avenue in the field of human–computer interactions [4]. Due to the convenient method of obtaining sEMG signals through electrodes attached to the skin’s surface, it has been widely used in various fields, including medical diagnosis, robot control, and gesture recognition. As a result, sEMG has gained significant attention and has become a popular choice for researchers alike. Nowadays, the rapid advancement of human–computer interactions has led to the development of intelligent prosthetics, which offer promising solutions for disabled people to enhance their daily lives. The integration of surface electromyography (sEMG) into human–computer interactions has significantly enhanced the functionality of intelligent prosthetics, allowing for precise gesture recognition. Advances in signal processing and adaptive learning technologies improve the system’s adaptability across different users, addressing challenges of signal variability and enhancing control accuracy for prosthetic devices [5]. At present, there are two widely used methods for gesture recognition: machine learning (ML) and deep learning (DL). Typically, gesture recognition is divided into four phases: signal acquisition, signal pre-processing, feature extraction, and signal classification. Among them, feature extraction engineering is the most important one, which directly impacts the recognition outcomes. The variability of sEMG data between individuals and the substantial noise in the signal make feature extraction a challenging task [6]. Numerous researchers are endeavoring to investigate novel approaches for extracting sEMG features [7]. Traditional machine learning usually needs to extract sEMG features and feed them to the classifier manually. In contrast, deep learning can autonomously capture and identify critical features [8], thereby enhancing performance effectively. Nowadays, applying deep learning for gesture recognition is quite widespread. A brief review of these two methods is outlined below.

The sEMG features can be categorized into time domain (TD), frequency domain (FD), and combinations of time domain and frequency domain [9]. For traditional methods, feature extraction is one of the most important issues affecting the classification results. In the literature, statistical algorithms, Fourier or wavelet transform empirical mode decomposition, and different techniques are applied for feature extraction. The combination of different features from the time domain, frequency domain, or both can be used as the input of the classifier. Machine learning classifier approaches include support vector machines (SVM) [10], random forest (RF) [11], and k-nearest neighbors (KNN).

Pizzolato et al. [12] extracted the time domain and frequency domain features within the 4 NinaPro datasets, and the hand gesture recognition results were approximately 74%. Altimemy et al. [13] utilized LDA and SVM methods with the time-domain feature Auto-Regressive (AR) to classify the hand gestures. Karnam et al. [14] proposed using a refined K-nearest neighbors (KNN) classifier in conjunction with ensemble energy features of the sEMG signal for hand activity classification, which achieved the highest accuracy of 88.8%. Aslan et al. [15] performed a Support Vector Machine (SVM) gestures classifier after extracting features with Distribution Entropy (DisEn) and Normal Cumulative Distribution Function (NCDF) methods. Recently, Fatimah et al. [16] decomposed the raw sEMG signals into a Fourier intrinsic band to extract statistical features for classification with SVM and KNN. As shown above, despite the remarkable performance of machine learning, the features were extracted manually, which may have resulted in the loss of some useful information related to the movement. Wang et al. [17] extracted six different time-domain features and chose the Linear Discriminant Analysis (LDA) classifier as a recognition algorithm, achieving an accuracy rate of 98.4%.

Since the successful application of deep learning in image recognition, researchers have attempted to extend its use in gesture recognition and achieve outstanding performance. Deep learning is characterized by its multi-layer network structure and is known for its ability to learn intricate hierarchical representations from data automatically. This approach is especially suitable for complex tasks like target recognition and object detection.

Cheng et al. [18] used CNN to extract image features from the sEMG signal of the NinaPro DB1 dataset to complete the classification and attain 82.54% accuracy. Wang et al. [19] created a feature map using wavelet decomposition and subsequently trained it with a combination of Convolutional Neural Network (CNN), Long Short-Term Memory (LSTM), and an attention mechanism, resulting in an accuracy of 86.36% using the DB1 dataset. Jiang et al. [20] proposed a novel Residual-Inception-Efficient (RIE) model based on multiscale fusion convolution and channel attention mechanism for classifying the DB1 dataset, achieving 88.27% accuracy. Zefeng et al. [21] designed a network based on transformers called the temporal depth-wise convolutional transformer (TDCT), which was developed for recognizing sparse sEMG signals and testing on various datasets. Sahoo et al. [22] proposed a 1D CNN model with fewer trainable parameters to classify the 52 gestures in the NinaPro DB1 dataset effectively. This model operated in a real-time system and provided a fast response, reducing the average inference time to 0.258 ms. Zhang et al. [23] employed the Hilbert filling curve to process the sEMG signal, obtaining images representing the temporal and electrode domains from the sEMG. These images are used as inputs to the block and propose the Dual-View network to obtain an accuracy of 86.72%, tested on the NinaPro DB1 dataset.

According to the existing literature, it is evident that deep learning techniques have the potential to alleviate the workload for gesture recognition. However, there is still a long way to go before employing surface electromyography (sEMG) signals in real-time applications. The main contributions of this paper are as follows:

- Innovative Architecture: By integrating 1D CNN and Bi-LSTM layers, the model effectively captures both sequential and long-term memory patterns within the sEMG signals. This dual-stream approach provides a simpler structure with fewer parameters, resulting in higher accuracy and reduced inference time, making it suitable for real-time control applications.
- Enhanced Feature Extraction: The combination of 1D Conv and LSTM layers enables the extraction of deeper, more informative features from the sEMG data, significantly improving classification results.
- Superior Performance: Extensive experiments conducted on the DB1 dataset, encompassing 52 different gestures, demonstrate that our proposed method outperforms well-known models, including the transformer model. These results confirm the superior accuracy and efficiency of our dual-stream feature fusion model.

The rest of the paper is organized as follows: Section 2 summarizes the preliminary introduction of each module of the proposed network model, and Section 3 describes the methodology we proposed. Section 4 presents the experimental details, including the data set, processing, and network hyperparameters. Section 5 includes the results and discussion of the experiment. The conclusion and future scope are in Section 6.

## 2. Preliminary

### 2.1. 1D Convolution Neural Networks (1D CNN)

Traditional convolutional neural networks (CNNs) are commonly applied for processing image data, whereas 1D CNNs are utilized for sequentially organized data. EMG represents a signal that can be conceptualized as a recorded sequence over time.

**Figure 1:**
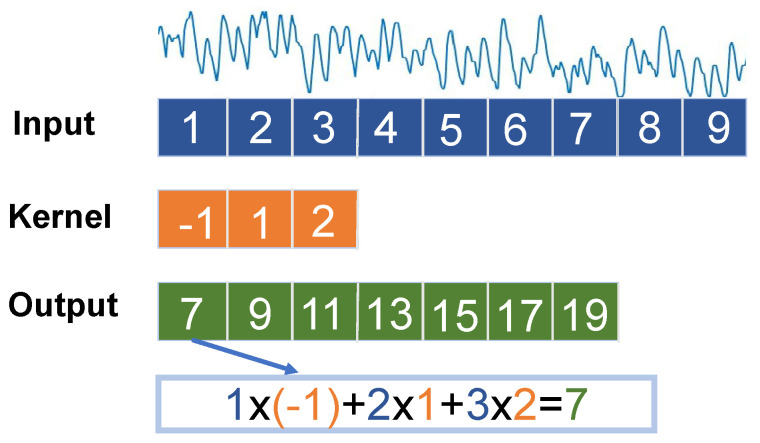
Illustraion of 1D CNN using an example of one channel signal.

The typical CNN architecture comprises convolution, pooling, activation, and fully connected layers. Convolution layers are responsible for learning patterns and features within the input data; an illustration of 1-D CNN is shown in Figure 1. Pooling layers can extract the most significant information to decrease the dimensionality of the data. As the number of convolution layers increases, the network will grow deeper. However, too many layers will cause a certain degree of overfitting, and the generalization of the network structure will decrease. However, the utilization of 1D CNNs reduces the dimensionality of the data compared to 2D CNNs, captures meaningful patterns, and enhances the computational efficiency of the models. Typically, the batch normalization (BN) layer is placed after the convolution layers to improve the speed of network training by mitigating the issues of vanishing or exploding gradients during the training period [6].

### 2.2. Long Short-Term Memory (LSTM)

**Figure 2:**
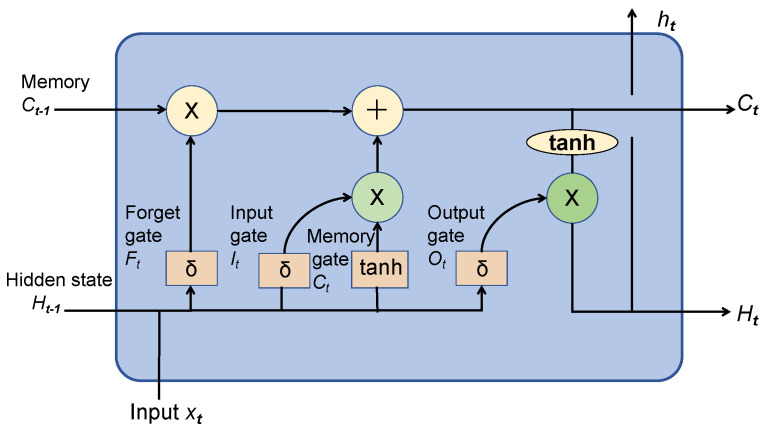
LSTM architecture diagram.

The Long Short-Term Memory (LSTM) model represents a distinct form of recurrent neural network (RNN) addressed to overcome the challenges related to vanishing gradients and gradient explosions. These structures consist of memory cells that are capable of retaining and modifying data over a period and are regulated by three gates: the input gate, the forget gate, and the output gate; as the architecture diagram of LSTM shows in Figure 2, this process is executed through the six equations given below [24]. In the Long Short-Term Memory (LSTM) model, a control gate is utilized to retain the current hidden state within a memory cell, taking into account previously hidden states [25]. To improve the processing capacity of LSTM networks when dealing with intricate data, researchers began to employ stacking multiple LSTM and bidirectional LSTM layers.

(1) $${\tilde{c}}_{t}=\tan h\left(W_{c}x_{t}+W_{c}x_{t-1}+b_{c}\right).$$

(2) $$i_{t}=\delta \left(W_{i}x_{t}+W_{i}h_{t-1}+b_{i}\right).$$

(3) $$f_{t}=\delta \left(W_{f}x_{t}+W_{f}h_{t-1}+b_{f}\right).$$

(4) $$o_{t}=\delta \left(W_{o}x_{t}+W_{o}h_{t-1}+b_{o}\right).$$

(5) $$c_{t}=f_{t}*c_{t-1}+i_{t}*{\tilde{c}}_{t}.$$

(6) $$h_{t}=o_{t}*\tan h\left(c_{t}\right).$$

In the formula, $i_{t}$ represents the input gate, $f_{t}$ represents the forget gate, $o_{t}$ represents the output gate, δ represents the sigmoid function, $w_{x}$ represents weight for the respective gate (*x*) neurons, $h_{t-1}$ represents the output of the previous LSTM block (at timestamp *t*−1), $x_{t}$ represents the input at the current timestamp, and $b_{x}$ represents the biases for the respective gates (*x*).

The bidirectional LSTM model employs two directions of sequence processing: forward (from beginning to end) and backward (from end to beginning). This method significantly increases the volume of data the network can access, making it a robust tool for modeling the sequential dependencies between words and phrases in both directions of the sequence. Additionally, it is beneficial for the entity process.

### 2.3. Concatenation

The concatenation layer is frequently employed in CNN for two primary purposes. One approach is to generate an abundant feature map by merging the outputs from multiple convolution layers. This technique is exemplified in the renowned ResNet architecture, where residual blocks employ concatenation to capture more complex feature representations [26]. Additionally, concatenation is utilized to create multi-scale feature maps by integrating convolution layers with diverse kernel sizes, enabling the acquisition of various information scales. This approach, essential for obtaining information at different scales, is particularly prevalent in tasks related to object detection and semantic segmentation.

## 3. Methodology

**Figure 3:**
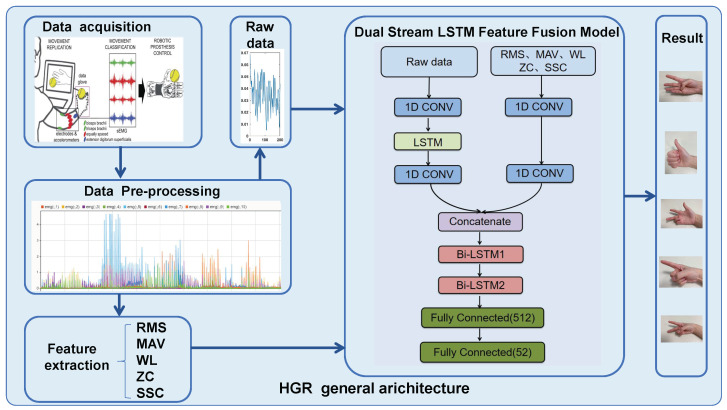
HGR diagram depicts steps included in the proposed dual stream LSTM feature fusion architecture (Data acquisition picture from “Atzori 2014 [28]”).

In this paper, the objective is to map the features of the EMG signal to classify hand gestures. The HGR (hand gesture recognition) general architecture is shown in Figure 3. First, the EMG signal was filtered, normalized, and segmented. Then, five time-domain features were extracted from the segmentation and a dual stream of the raw data and five time-domain features were fed into the 1-D CNN layer to capture the spatial and short-time relationships, into a concatenation layer to join the features in one dimension, and then into two Bi-LSTM layers to learn long-term memory information for the classification. Features from handcrafted and deep learning techniques both help obtain different types of encoding information to distinguish gestures, and a hybrid feature set can provide complementary information that can improve the classification results [27].

### 3.1. Five-Time-Based Feature Extraction

The paper focuses on extracting five time-domain features for concatenation with the raw signal, forming a dual stream of input features for subsequent processing. The feature set comprises $f_{MAV}$, $f_{ZC}$, $f_{SSC}$, $f_{WL}$, and $f_{RMS}$, and their details are presented in Table 1. The feature set is computed by each segment of a given trial, partitioned based on the window size.

As the number of samples for every trial is 500, the window size in this paper is 200 ms (20 sample points) and 50 ms (5 sample points). Take the window size of 200 ms to explain how creating the feature set works. The number of every time-domain feature $N_{f}$ in each trial is 25 ($N_{T}$/sample points of one window size). Because the dataset has 10 channels, the feature shape is 25 × 5 × 10 in each trial. The structure is constructed to facilitate its subsequent integration with the raw data, which plays a critical role in the concatenate step.

### 3.2. Network Model

**Figure 4:**
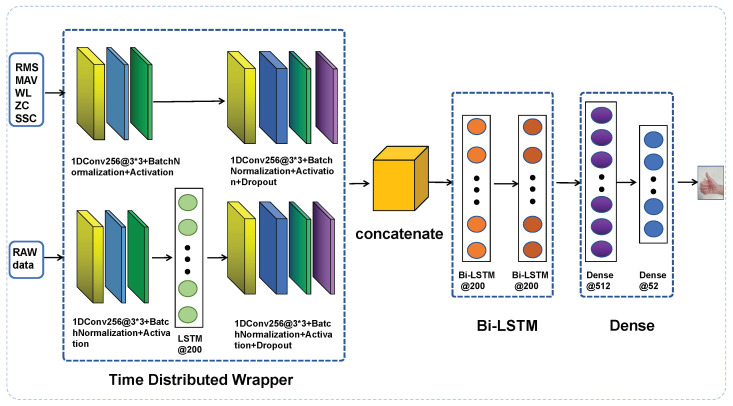
Framework of the dual stream model based on LSTM and CNN architecture.

The framework contains a dual stream that processes the five time-domain features and raw data independently, as the architecture shows in Figure 4. The dual stream model features and raw model incorporate 1D CNN layers, batch normalization, activation, and dropout layers. These layers are encapsulated in a time-distributed manner, processing the stream at each time step of the data independently. Once the processing is complete, their outputs are aggregated using the concatenate layer. Subsequently, the combined output is passed through the bidirectional LSTM and dense layers.

To capture different relevant features from EMG signals, a dual stream of 1D CNN layers in a time-distributed wrapper is utilized to process five time-domain features and raw data. The key distinction lies in the incorporation of an LSTM layer between the two 1D CNN layers, enabling the capture of the sequential nature of the raw signal. The inclusion of the LSTM layer in the raw data is motivated by its ability to capture sequential information, which corresponds to the EMG signal, which is inherently a sequence. Each stream of convolution layers can concentrate multiple aspects of the data, acquiring diverse representational features. Following the convolutional layers, a concatenate layer is employed to combine the stacks of feature maps along a novel dimension, thereby enhancing the data into an extended feature vector that captures a broader range of data characteristics. The results of the concatenate layer are subsequently fed into two bidirectional Long Short-Term Memory (LSTM) layers, which are designed to capture temporal dependencies in both forward and backward directions. Intuitively, it is widely recognized that Bi-LSTM can encode long-term temporal information more effectively than uni-directional LSTM [24].

**Figure 5:**
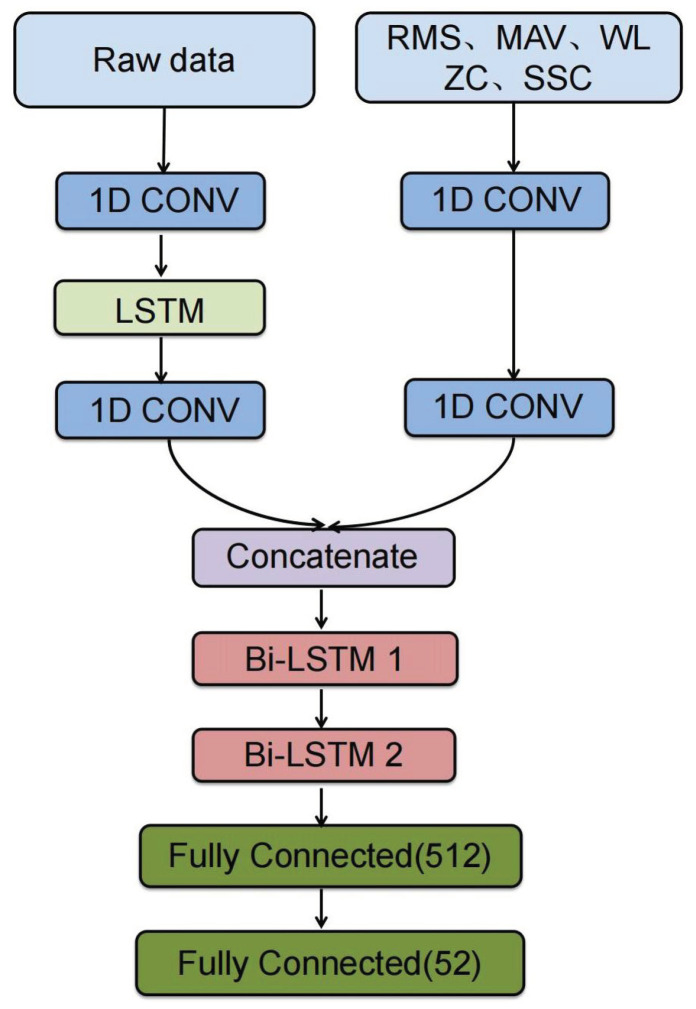
The block diagram of dual stream model.

The output of the two Bi-LSTM layers is transformed into a one-dimensional tensor and subsequently fed into a fully connected layer (FC) equipped with activation functions, dropout, and batch normalization to standardize the outputs of the dense layer. The final layer consists of a dense layer with a softmax activation function, designed for multi-class classification; in our case, the output is 52 hand gestures, as shown in the block diagram shown in Figure 5.

In DB1, the total number of sEMG patterns is $N=S\;*\;N_{a}\;*\;R$, so in this paper N=27∗52∗10=14,040, *S* is the subject numbers; $N_{a}$ represents the numerical hand gesture; while R denotes the repetition times for each participant’s execution of each gesture. In this paper, the number of samples for each trial is $N_{T}$.

$N_{T}=N_{s}*T$, $N_{T}$ is the number of samples in every trial, *T* is the number of values in one trial of duration, and $N_{s}$ is the sampling rate (samples/s). In our case, $N_{T}=500$, according to the segment size, $N_{T}$ is converted into pandas data frames with TensorFlow from beginning to end. The tensor shapes at each step of the outlined network layer are given in Table 2 (window size of 200 ms).

## 4. Experiments

The experiment is carried out on Intel(R) Core (TM) i7-3770K CPU @3.50 GHz (8 CPUs). The proposed approach is implemented by the TensorFlow 2.2.0 framework, coupled with Python 3.8. This segment of this research utilizes the benchmark Ninapro DB1 dataset, which is accessible from the official Ninapro repository [35].

### 4.1. Dataset Description

**Figure 6:**
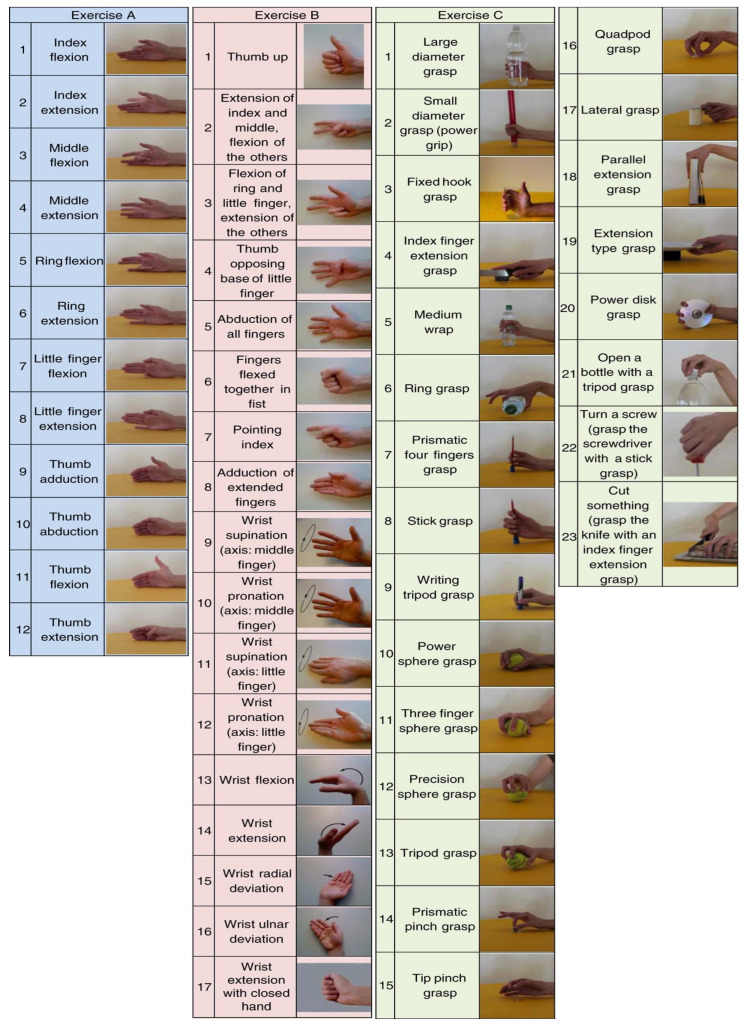
The 52 hand movements in the NinaPro DB1 Dataset [28].

The Ninapro DB1 dataset [28,36,37] comprises 52 distinct gestures (tag No. 1–52), as shown in Figure 6 from 27 subjects (20 males and 7 females, aged 28 ± 3.4-year-old). The gestures can be categorized into three distinct groups of movements: (a) individual finger flexion and extension movements; (b) simultaneous flexion and extension movements of multiple fingers, as well as the wrist; and (c) grasping objects, as shown in Table 3. There are 52 labels and corresponding gesture categories, as shown in Figure 6. The sEMG data are captured at a sampling rate of 100 Hz with ten Otto Bock electrodes with filter cutoff frequencies set at 90 Hz and 450 Hz [28]. Each participant performs a single iteration of each movement for 5 s, followed by a 3-s rest period; this cycle is repeated 10 times.

### 4.2. Data Processing

The key information in surface electromyography (sEMG) is primarily concentrated within the frequency range of 20 to 200 Hz [38]. Before integrating the sEMG raw signal, it is imperative to remove various types of noise, including those from power lines and environmental noise, as they are typically present in the signal. Before the release of the Ninapro database, the signals were synchronized using high-resolution timestamps. Errors in the synchronization of each movement were corrected, and a 50 Hz filter was employed to mitigate line noise [12,28].

In the pre-processing stage (as shown in Figure 3), initially, raw sEMG data are filtered by a Butterworth high-pass filter to diminish the noise interference. Afterward, the data are standardized by utilizing the Z-score method (as in Equation (7)) to minimize the impact of data variations.

(7) $$z=\frac{x-\mu }{\delta }.$$

*z* is the normalized value, *x* is the original value, μ is the mean value, and δ is the standard deviation.

**Figure 7:**
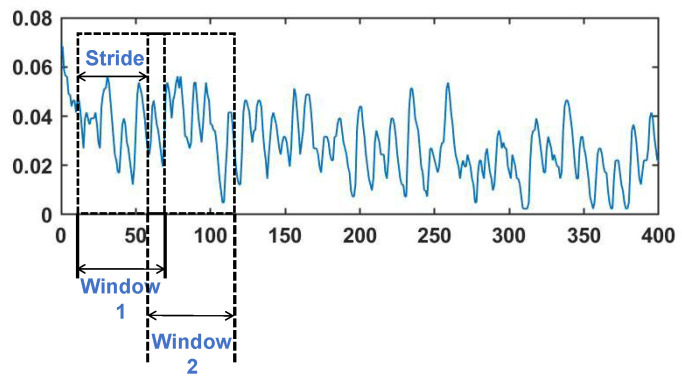
Sliding window segmentation of the sEMG data.

A window size is selected for data segmentation, typically to facilitate real-time control. Previous studies have indicated that a delay of 300 ms fulfills the requirements [39]. The stride parameter defines how much advancement occurs with each window size. In our research, the window sizes of 200 ms and 50 ms with the same stride step were chosen; Figure 7 shows a single channel of sEMG signal employing a sliding window technique.

### 4.3. Network Settings and Model Training

The architecture is executed with the Tensor Flow 2.2.0 framework in conjunction with Python 3.8 and Matlab 2022b.

The specific details of each convolutional layer, kernel size, LSTM unit, and dense layer are given in Table 2. Initially, the learning rate is set to 0.0001 in the first 70 epochs, followed by a step decay rate of 0.1 drops to enhance the model’s performance. The batch size is set at 64. The specific hyperparameters used to optimize a better result for this research are concluded in Table 4.

### 4.4. Loss and Optimization

The loss function is used to articulate the disparity between the anticipated and observed values, serving as a measure metric to assess the performance of the model. For classification tasks, the cross-entropy loss is commonly employed; the mathematical formula is given in (8), where M denotes the batch size, and $\;y_{i}$ and ${\hat{y}}_{i}$ represent the anticipated and observed label, respectively.

(8) $$Loss=-\sum_{i=1}^{M}y_{i}\cdot \log{\hat{y}}_{i}.$$

Optimizers are algorithms or techniques employed to minimize the error function (loss function) or to enhance the productivity efficiency [40]. The Adam optimization algorithm incorporates exponentially weighted averages to enhance the gradient descent process and functions as an extension of the conventional gradient descent optimization algorithm [41]. The Adam optimization is preferred for its enhanced ability to generalize. Hence, this paper selects Adam optimization for further investigation.

### 4.5. Network Evaluation

The dual-stream model is trained and validated separately on distinct data. Accuracy is the predominant assessment criterion for the classification tasks and is evaluated as follows:

(9) $$Accuracy=\frac{Number\;of\;correctly\;classified\;trails}{Total\;number\;of\;trails}\times 100\%$$

The confusion matrix provides a comprehensive representation of predictive performance. So, in this research, accuracy is utilized to evaluate the effectiveness of the proposed approach for sEMG recognition and present a summary using a confusion matrix.

## 5. Results and Discussion

To validate the effectiveness of the dual stream feature fusion model, the following experiments are performed: (a) various input shapes; (b) short-circuit the input stream of five time-domain features, remove the concatenation layer, and remove the LSTM layer between the 1D conv layer in the raw data. In the following, Model A represents without a dual stream of input; the window size is 200 ms. Model B represents with a dual stream of input; the window size is 50 ms. Model C represents with a dual stream of input; the window size is 200 ms.

### 5.1. Comparison of Various Input Shapes

In this paper, the analysis is carried out with two distinct input shapes as shown below for the DB1 dataset. The findings demonstrate a high degree of comparability between the results obtained with varying window sizes.

**Figure 8:**
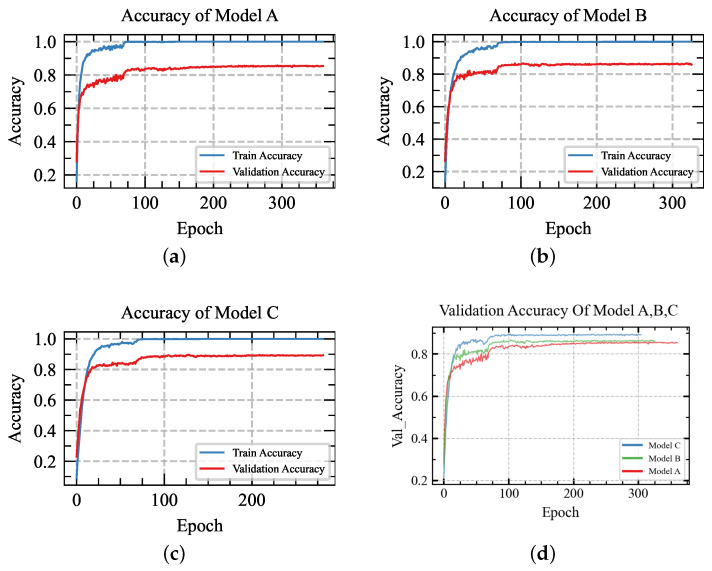
Comparing accuracy across models A, B, and C.

**Figure 9:**
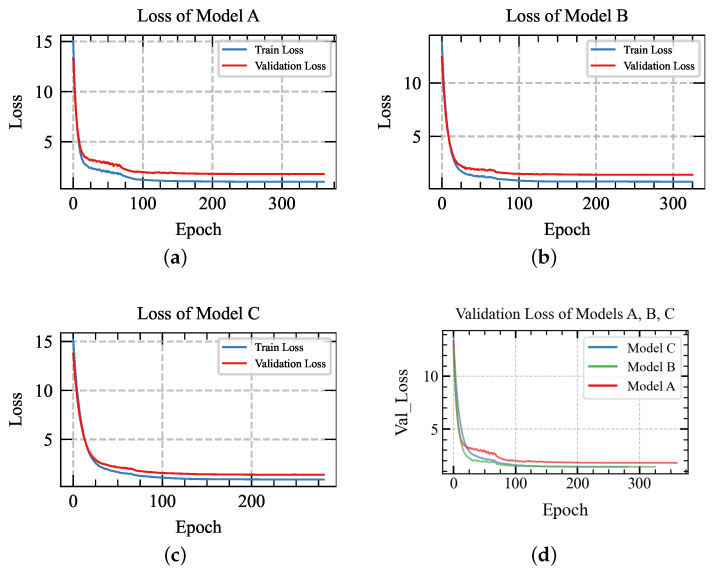
Comparing loss across models A, B, and C.

The graphs display the loss and accuracy curves over epochs for training and validation using various window sizes, as shown in Figure 8a,c and Figure 9a,c. As depicted in the illustration, the network effectively accomplishes the classification tasks and achieves outstanding accuracy. For the window size of 200 ms, the training accuracy can reach 100%, the validation accuracy can reach 89.66%, and each gesture inference time is 87.6 ms. In comparison, for the window size of 50 ms, the validation accuracy can reach 85.291%, and the inference time is 70.4 ms. Upon reaching 100 epochs, the loss of different window sizes can be balanced. This model illustrates the methods of concatenating the features extracted from time-domain features and raw data using 1D convolutional and LSTM layers to capture pertinent information from the sEMG signal. The classification accuracy shows a strong correlation with the window size, with an appropriate window size impacting the recognition result.

### 5.2. Comparison without Dual-Stream Stream Model

**Figure 10:**
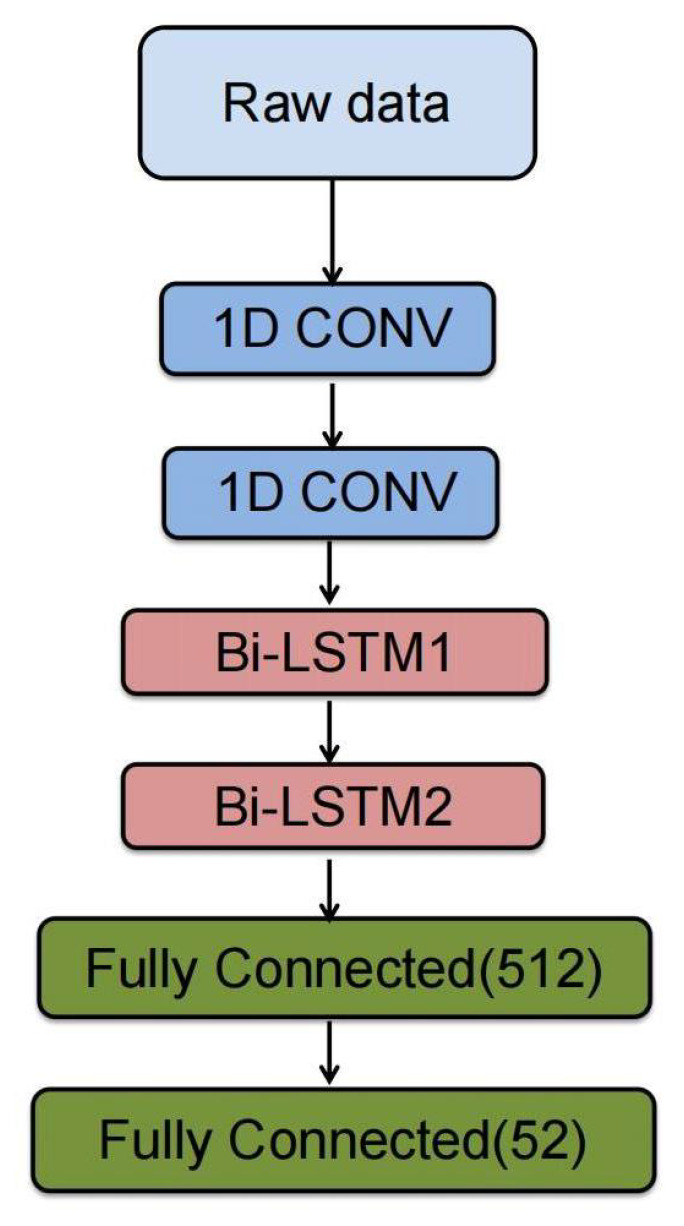
The block diagram without the dual stream model.

In this part, the experiment is carried out using the model as shown in Figure 10. This setup involves a single input stream of raw data, bypassing the additional five time-domain feature streams and the LSTM layer typically used in raw data analysis. Instead, a solitary 1D convolution layer is applied to extract features from the raw data for gesture recognition, as Figure 8b and Figure 9b show, the training accuracy can achieve 100% and the validation accuracy can achieve 86.69%, which is lower than that of the dual stream model; additionally, the loss gets balanced, and the inference time gets optimized to 108 ms. These results underscore the potential of our proposed model to enhance gesture recognition results further.

**Figure 11:**
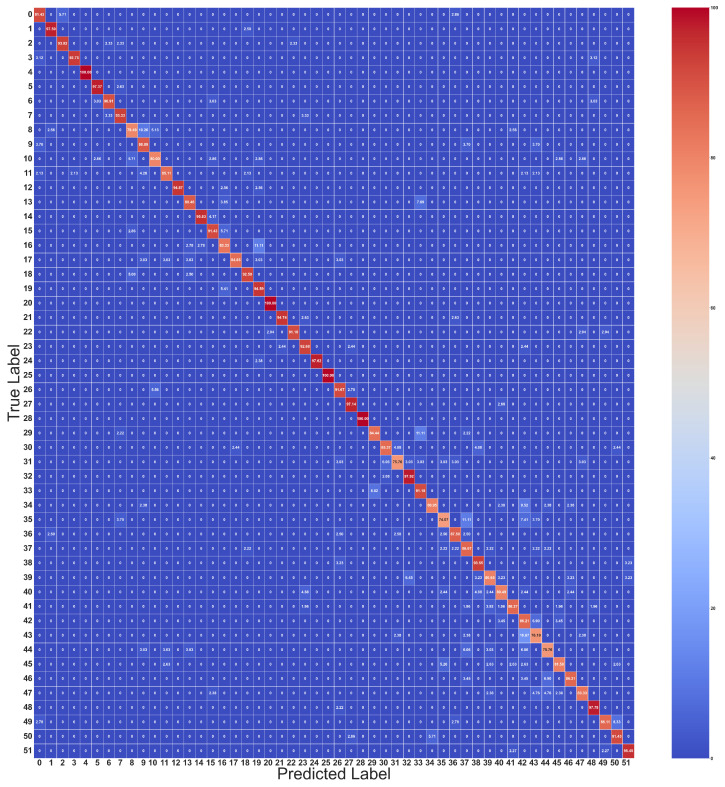
Confusion matrix for dual stream feature fusion classifier. (Diagonal data: 0: 91.43, 1: 97.50, 2: 93.02, 3: 93.75, 4: 100, 5: 97.37, 6: 90.91, 7: 93.33, 8: 79.49, 9: 88.89, 10: 80.00, 11: 85.11, 12: 94.87, 13: 88.46, 14: 95.83, 15: 91.43, 16: 83.33, 17: 84.85, 18: 92.50, 19: 94.59, 20: 100, 21: 94.74, 22: 91.18, 23: 92.68, 24: 97.62, 25: 100, 26: 91.67, 27: 97.14, 28: 100.00, 29: 84.44, 30: 85.37, 31: 75.76, 32: 97.92, 33: 91.18, 34: 80.95, 35: 74.07, 36: 87.50, 37: 86.67, 38: 93.55, 39: 80.65, 40: 80.49, 41: 86.27, 42: 86.21, 43: 76.19, 44: 75.76, 45: 81.58, 46: 86.21, 47: 83.33, 48: 97.78, 49: 86.11, 50: 91.43, and 51: 95.45).

**Figure 12:**
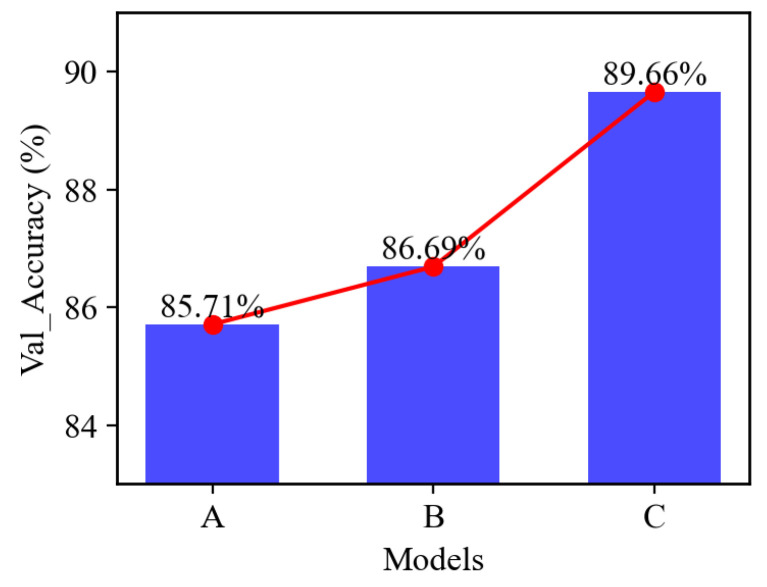
Validation accuracy comparison of models A, B, and C.

The confusion matrix is employed to evaluate the performance of a classification network. Through the confusion matrix, the classifier accuracy of each gesture is shown. In the DB1 dataset, the similarity between several gestures can degrade classification accuracy. As shown below, Figure 11 is the confusion matrix of the dual stream model with a 200 ms window size, and Figure 8d, Figure 9d and Figure 12 show the overall trend of accuracy, loss, and results in comparison. The figure highlights that the dual-stream structure not only achieves higher accuracy results but also benefits from shorter inference time. This enhancement is largely attributed to the unique feature fusion method, which collects feature engineering using 1D conv layers from time-domain features and the raw signal. This method enables the capture of more deep features but also obtains short sequence features from sEMG signals. These engineered features are then fused and processed through the Bi-LSTM layers to capture long-term sequence feature information. This structural combination is particularly effective for long-sequence signals such as sEMG signals, which can significantly boost classification accuracy and shorten the inference time.

### 5.3. Validation on Ninapro DB2 Dataset

This study utilizes the Ninapro DB2 dataset [28] to perform a validation of the proposed model; this dataset includes 40 subjects performing 49 different hand and finger movements, plus a rest position. The sEMG data for the gesture movements were collected with 12 channel electrodes, at a sampling rate of 2000 Hz. The movements are performed for six repetitions lasting five seconds, followed by a three-second rest.

**Figure 13:**
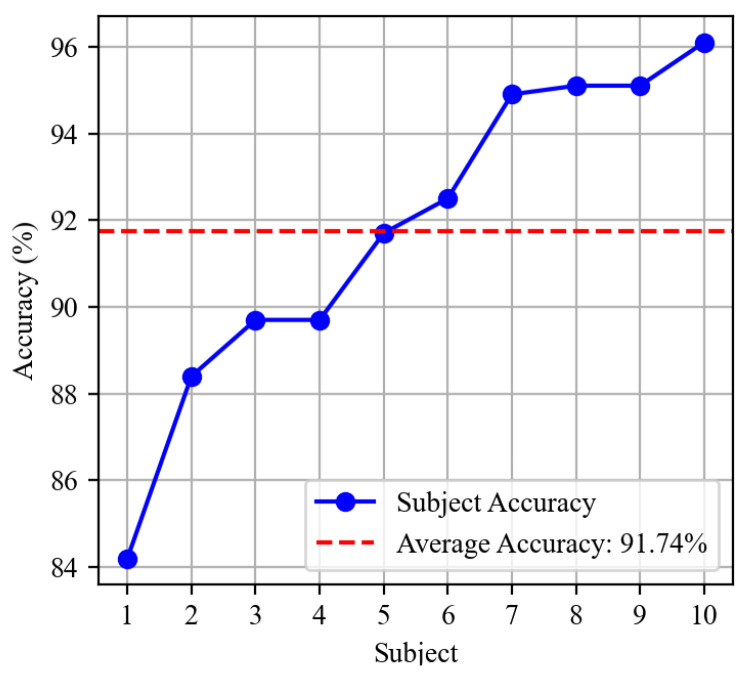
Validation accuracy of 10 subjects from DB2 dataset.

In this research, 10 out of 40 subjects from the DB2 dataset were randomly selected to conduct a subject-wise experiment. All procedures followed those established for the DB1 dataset, including pre-processing, segment size, and fine-tuning hyperparameters for the classifier. The proposed model undergoes evaluation under a subject-wise analysis framework [24]: In this scheme, the dual stream model is individually trained and validated using data specific to each subject. This approach is intended to closely resemble practical application, where a model would be personalized and adapted to an individual user. By focusing on subject-wise data for both training and validation, the model can effectively learn the unique characteristics and variations of each person’s EMG signals, leading to more accurate and reliable gesture recognition for that individual. The overall classification accuracy is calculated by averaging the results across all subjects to evaluate the model’s performance comprehensively. Detailed results are presented in Figure 13.

The proposed model excels with various datasets; the accuracy of the subject-wise model exceeds 84%, with peaks at 96%. The average accuracy across all subjects reaches 91.74%.

### 5.4. Comparison of Results with Previous Works

Recently, numerous investigations were conducted on the utilization of EMG signals for gesture recognition and achieved good results. In this research, the proposed model is assessed for accuracy in comparison to recent research results that also utilize the same Ninapro DB1 dataset. The details of the results are given in Table 5. Notably, most current deep learning approaches for hand gesture classification, including those incorporating CNNs, have achieved commendable results. Some of the researchers integrate CNN with other methods, surpassing networks that solely rely on CNN. Moreover, transformer-based networks employing the same features have pushed the classification accuracy to a new level, which is over 89%. If the inference times for all models listed in the table were available, such a comparison would be particularly valuable for real-time applications.

This study presents a framework designed to derive insights from EMG signals from multiple sides, including raw data and time-domain features. The proposed model significantly outperforms other models using the same dataset in terms of classification accuracy.

The enhanced performance of the dual-stream model can be attributed to several key points:

- The use of a 1D Convolution layer to simultaneously process features from two distinct streams—time-domain features and raw data—enables the capture of both spatial and temporal dependencies.
- A concatenated feature fusion approach serves as input to two bidirectional LSTM layers, which are engineered to recognize temporal patterns from both forward and backward directions.
- Within the raw data stream, an LSTM layer integrated with the 1D convolution captures the sequential nuances of the data, enhancing the model’s predictive accuracy.

## 6. Conclusions and Future Scope

### 6.1. Conclusions

This research introduces a novel methodology that employs a dual-stream convolution layer to derive features from both time domain characteristics and raw data. This technique effectively catches spatial and temporal dependencies in the data simultaneously, allowing the processed input to feed into bi-LSTM layers for enhanced hand gesture recognition. The model is evaluated on the benchmark dataset Ninapro DB1 and achieves a commendable accuracy of 89.66%, which surpasses the existing research methods utilizing the same dataset, with an inference time for each gesture of 87.6 ms. Furthermore, when validating a portion of the DB2 dataset, the model demonstrates considerable efficacy, with an average accuracy of 91.74% across ten subject-wise evaluations, underscoring the significant potential of the proposed model. The experimental results reveal that the dual-stream feature fusion model adeptly captures multi-scale information from both time-domain features and the raw signal. The integration of 1D convolution, LSTM layers, and dropout techniques leads to a notable improvement in gesture recognition performance utilizing the EMG signal.

### 6.2. Future Scope

Future initiatives will focus on refining the proposed algorithm to enhance the recognition of upper arm gestures when integrated with an exoskeleton, and on streamlining the network architecture to decrease inference times for application in real-time control systems. Additionally, the development of an online collection system for surface electromyography (sEMG) signals is underway to achieve the instantaneous analysis of human movement intention. The objective of this system is to facilitate real-time control for an exoskeleton to assist individuals with physical limitations. By employing advanced signal processing techniques, the sEMG signals can be translated into actionable commands for the exoskeleton, empowering the user to perform previously challenging movements. This technological innovation represents a significant advancement in biomechanics and holds promise to enhance the quality of life for diverse individuals.

## Figures and Tables

**Table 1:** Feature extraction method.

S/N	Features Description	Mathematical Expression
1	Root Mean Square (RMS): This is modeled as amplitude- modulated Gaussian random process that relates to constant force non-fatiguing contraction [29]	$\sqrt{\frac{1}{k}\sum_{n=1}^{k}{x_{n}}^{2}}$
2	Mean Absolute Value (MAV): This is an average of absolute value of the EMG signal in an analysis time window [30]	$\sum_{n=1}^{k-1}\left[f\left(\left|x_{n+1}-x_{n}\right|\right)\right]$
3	Waveform Length (WL): This is the aggregate length of the EMG waveform in an analysis window [31]	$\frac{1}{k}\sum_{n=1}^{k}\left|x_{n}\right|$
4	Zero Crossing (ZC): This refers to the number of times that the EMG signal changes from positive to negative or vice versa [32]	$ZC=\sum_{i=1}^{N=1}\left[sgn\left(x_{i}\times x_{i+1}\right)\cap \left|x_{i}-x_{i+1}\right|\geq threshold\right]$ $sgn\left(x\right)=\left\{\begin{array}{cc} 1 & ifx\geq threshold\\ 0 & \;\mathrm{otherwise} \end{array}\right.$
5	Slope Sign Change (SSC): This indicates frequency information of the EMG signal, as the number of times that the slope changes from positive to negative or vice versa [33,34]	$SSC=\sum_{i=2}^{N-1}\left[f\left[\left(x_{i}-x_{i-1}\right)\times \left(x_{i}-x_{i+1}\right)\right]\right]$ $f\left(x\right)=\left\{\begin{array}{cc} 1 & \;\mathrm{if}\;x\geq threshold\\ 0 & \;\mathrm{otherwise} \end{array}\right.$

**Table 2:** Tensor shape obtained during implementation-data aggregate scheme.

Layer	Tensor Shape of Feature	Tensor Shape of Raw Data
Input	(25, 5, 10)	(25, 20, 10)
Conv1D	(25, 3, 256)	(25, 10, 256)
LSTM	NONE	(25, 10, 256)
Conv1D	(25, 2, 256)	(25, 5, 256)
Flatten	(25, 512)	(25, 1280)
Concatenated	(25, 1792)	
Bi-LSTM	(25, 200)	
Bi-LSTM	(25, 200)	
Flatten	(25, 200)	
Dense 1	(25, 512)	
Dense 2	(25, 52)	

**Table 3:** The label of gesture activity on the NinaPro DB1.

Label	Gesture Activity
1–12	Basic movements of the fingers [28].
13–29	Isometric, isometric hand configurations, and fundamental wrist movements [28].
30–52	Grasping and practical movements [28].

**Table 4:** Tuned hyperparameters for the dual stream LSTM feature fusion classifier.

Layer	Value
Batch size	64
Learning rate	0.0001
Optimizer	Adam $\beta_{1}=0.9$, $\beta_{2}=0.999$
Activation	Tanh & Relu & softmax
kernel_regularizer	0.01
Batch normalization	epsilon = 1× 10^−5^, momentum = 0.9
Number of epochs	500

**Table 5:** Detailed comparison across various networks.

Classifier (%)	Window Length (ms)	Dataset	Features	Accuracy
Random Forests [28]	200	DB1	RMS, MAV, WL, ZC, SSC, and Histogram	75.32%
KNN [14]	-	DB1	MAV, Temporal Segment Energies (TSE) and the Spectral Band Energies (SBE)	88.80%
MsCNN [42]	200	DB1	-	85.00%
EvCNN [43]	200	DB1	-	81.40%
multi-view CNN [44]	200	DB1	-	88.20%
CFF-RCNN [45]	250	DB1	-	88.87%
1D CNN [22]	150	DB1	-	78.95%
Transformer [21]	100, 150 and 200	DB1	MAV, ZC, SSC, WL, and RMS	89.43%
**Proposed**	50 and 200	DB1	MAV, ZC, SSC, WL, and RMS	**89.66%**
	200	DB2		**91.74%**

## Data Availability

The dataset can be accessed at http://ninapro.hevs.ch (accessed on 30 May 2024).

## Abbreviations

The following abbreviations are used in this manuscript:

sEMG	Surface electromyography
CNNS	Convolutional neural networks
LSTM	Long Short-Term Memory
HGR	Hand gesture recognition
